# A systematic review of studies on stress during the COVID-19 pandemic by visualizing their structure through COOC, VOS viewer, and Cite Space software

**DOI:** 10.3389/fpsyt.2024.1297112

**Published:** 2024-01-25

**Authors:** Liyun Lu, Guiping Liu, Yanhua Xu, Jinxiu Jiang, Zizi Wei

**Affiliations:** ^1^ College of Teacher Education, Nanjing Normal University, Nanjing, China; ^2^ School of Geography and Environment, Jiangxi Normal University, Nanchang, China; ^3^ College of Teacher Education, East China Normal University, Shanghai, China

**Keywords:** COOC analysis, VOS viewer, Cite Space, stress during COVID-19, bibliometric analysis

## Abstract

**Background:**

The COVID-19 epidemic generated different forms of stress. From this period, there has been a remarkable increase in the quantity of studies on stress conducted by scholars. However, few used bibliometric analyses to focus on overall trends in the field.

**Purpose:**

This study sought to understand the current status and trends in stress development during COVID-19, as well as the main research drives and themes in this field.

**Methods:**

2719 publications from the Web of Science(WOS) core repository on stress during COVID-19 were analyzed by utilizing Co-Occurrence (COOC), VOS viewer, and Cite Space bibliometric software. The overall features of research on stress during COVID-19 were concluded by analyzing the quantity of publications, keywords, countries, and institutions.

**Results:**

The results indicated that the United States had the largest number of publications and collaborated closely with other countries with each other. University of Toronto was the most prolific institution worldwide. Visualization and analysis demonstrated that the influence of stress during COVID-19 on the work, life, mental and spiritual dimensions is a hot research topic. Among other things, the frequency of each keyword in research on stress during COVID-19 increased from 2021 to 2022, and the researchers expanded their scope and study population; the range of subjects included children, nurses, and college students, as well as studies focusing on different types of stress, and emphasizing the handling of stress.

**Conclusion:**

Our findings reveal that the heat of stress research during COVID-19 has declined, and the main research forces come from the United States and China. Additionally, subsequent research should concern more on coping methods with stress, while using more quantitative and qualitative studies in the future.

## 1 Introduction

Since December 2019, the COVID-19 pandemic, generated by Severe Acute Respiratory Syndrome Coronavirus 2 (SARS-CoV-2), has impacted people and systems around the globe. Due to the high transmission and mortality rates of the disease, as well as the continuous media coverage, it has attracted a significant attention, thereby elevating stress levels and anxiety, and causing social panic (1–4). When people are in a state of extreme stress, they tend to experience a series of psychological problems such as anxiety and depression (5–9). Moreover, the forceful variation in daily lifestyle due to the pandemic enhances the development of poor mental health conditions and behaviors, such as alcohol abuse, lack of exercise, and insomnia (10–13). Therefore, research related to stress during the COVID-19 period plays an essential role for keeping individuals in good mental health.

Current research on stress during COVID-19 has focused on four primary areas: causes of stress, influential elements, impacts on individuals, and relief measures. First, on the formation causes of stress during COVID-19, the COVID-19 lockdown enhanced changes in human lifestyles, thereby increasing the incidence of anxieties, stresses and depressions and creating a wide range of stresses (14–17). For instance, for remote workers, the COVID-19 lockdown rendered work technologically more stressful (18–21). Second, scholars investigated the influence of stress during COVID-19 on individual, institutional, and societal levels (22–25). Sagherian et al. demonstrated that optimizing nursing schedules and practices reduced stress among nursing staff (26). Third, regarding the human impact of stress during COVID-19, stress during COVID-19 affects the daily life of different population groups (college students, pregnant women, and healthcare workers, among others), and enhances mental health problems (27–31). For example, the study found that the COVID-19 epidemic negatively impacted the psychological well-being of undergraduate medical students, with increased incidence as well as levels of anxiety and stress (32–35). Fourth, in terms of methods of stress relief during COVID-19, scholars have explored ways to relieve stress, such as improving lifestyle and adopting positive health behaviors (36–40). Mattioli et al. demonstrated that isolation is associated with stress and depression, and that global action must be engendered to support healthy diet and physical activity, thereby promoting good lifestyles (41). In addition, utilizing external group music therapy with group speech therapy during COVID-19 lead to better stress management (42).

Despite many studies on stress during COVID-19, most have specific inclinations, and lack a holistic perspective and a systematic analysis of the literature in this field using bibliometric methods. For example, Khanal et al. focused on factors related to anxiety, depression, and insomnia within the healthcare workforce during the pandemic (43), whereas Tabur et al. focused on job stress, social support, and turnover intention among the same group (44). Even the review literature mostly focused on specific aspects of different population groups (older adults, adolescents, pregnant women, college students, and healthcare workers), and research methods used are mostly meta-analyses that waives the application of bibliometric methods for systematically analyzing the literature (45–47). Safi-keykaleh et al. utilized meta-analysis to assess the incidence of postpartum depression during COVID-19 (48). In addition, Chutiyami et al. utilized meta-analysis to synthesize the overall psychological well-being of healthcare professionals during COVID-19 (49). Therefore, it is important to perform a systematic analysis of stress research during COVID-19 using bibliometrics to evaluate the present state of application and developmental trends in the field.

Moreover, bibliometric analysis is a valuable tool for quantitative assessment of various parameters associated with scientific publications in a given area, revealing insights into prevalent research topics, developmental trends, critical researchers, and scientific organizations (50). However, articles in this area of stress during COVID-19 waived the method of bibliometric to analyze literature. Therefore, we employed an innovated method of literature review by a systematic review using COOC, VOS viewer and Cite space software as research tools. Besides, the use of COOC and VOS viewer allows visual analysis and mapping of scientific knowledge to demonstrate the structure, evolution, cooperation and other relationships in the field (51). Moreover, Cite Space software presents the structure of knowledge in a specific research area and summarizes trends in the development and evolution of that domain (52). A systematic literature review is a way of locating, evaluating and synthesizing evidence, as well as an orderly way of reviewing and summarizing research findings (53). This makes it significant during COVID-19. For instance, the analyze of the literature can characterize the overall magnitude of stress and depressive symptoms caused by the COVID-19 outbreak, which will lead policymakers to more judiciously measure the burden caused by similar public health events (54, 55). Thus, it is vital to conduct systematic reviews of previous relevant publications on stress during COVID-19 in order to realize the current state of application and trends of stress during large-scale public health events. Specifically, the six following research questions were proposed:

RQ 1: What are the dynamics of literature issuance in the field of stress during COVID-19 epidemic according to volume and frequency of citations?
RQ 2: Which countries, institutions and authors are more prolific based on the volume of publications?
RQ 3: What is the collaboration between countries, institutions and authors?
RQ 4: Based on citation frequency, which articles are important in the area of stress during COVID-19?
RQ 5: What are the research topics of interest in the field of stress during COVID-19?
RQ 6: What is the trend of research evolution in the field of stress during COVID-19?

## 2 Methodology

### 2.1 Data collection and pre-processing

**Figure 1:**
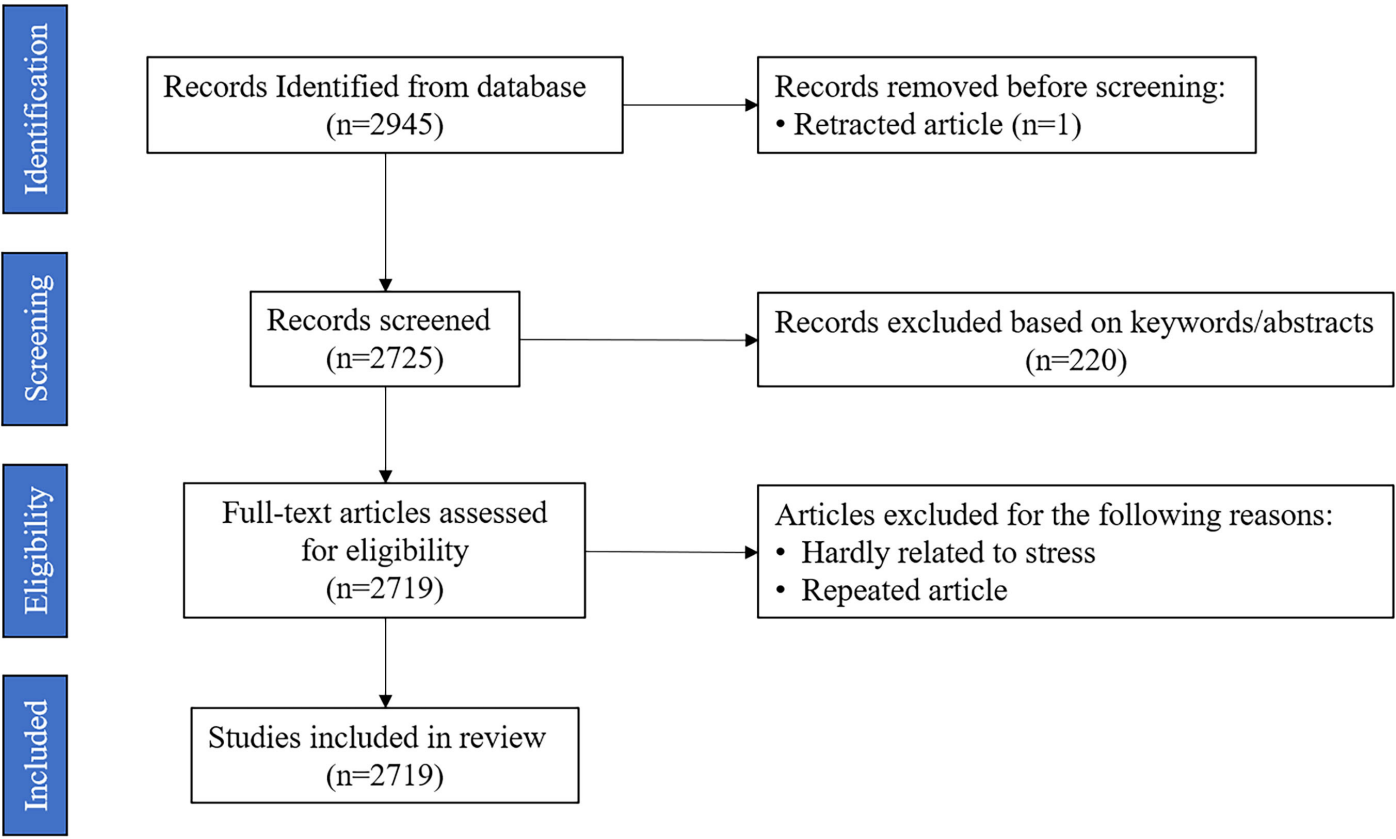
Flowchart of the data collection.

This study was implemented on June 15, 2023, where data was collected from relevant literature in Web of Science (WOS)—a comprehensive, multidisciplinary, core journal citation indexing database—which is a common tool for bibliometrics. The data utilized in this study are derived from the WOS Core Collection, with a search date of June 15, 2023. To ensure the accuracy of the search, this study followed the PRISMA guidelines for literature collection, and constructed the search formula TS = “Stress” AND “During COVID-19”, the language was “English”, the literature types were “Dissertation”, “Online publication”, “Review paper”, and the time was not limited; this study obtained 2,946 papers (see **Figure 1**). After manual screening, 2,719 valid papers were retained.

Data preprocessing was the first part of data analysis. The following data preprocessing steps were implemented in this study. First, the researcher exported the full records of retrieved articles and cited references in plain text format. Second, key elements of all articles (year, citation frequency, title, author keywords, additional keywords, journal, author, institution, and country) were extracted into.elsx files. Third, duplicate articles were removed. Fourth, missing elements were added in the articles. Fifth, irrelevant terms were screened and excluded, and synonyms were merged. Sixth, the format of the elements was standardized by removing extra spaces and punctuation.

### 2.2 Research tools and data processing methods

Visualization helps to analyze, and explore large-scale and complex data. In this study, COOC, VOS viewer and Cite Space software were used as research analysis tools. First, COOC software was used to count the valid literature. Second, VOS viewer and Cite Space software were used to analyze the partnerships, and key themes of the research topic of stress during COVID-19.

Part I: COOC Analysis Procedure

In the WOS database, 2,946 articles were retrieved from 2020 to 2023 using a search formula, and 2,719 articles meeting the inclusion criteria were screened after data preprocessing using COOC software. Afterwards, the COOC software was used to analyze the valid literature, and the chronological volume and evolutionary trend maps were generated.

Part II: VOS viewer and Cite Space analysis program

**Figure 2:**
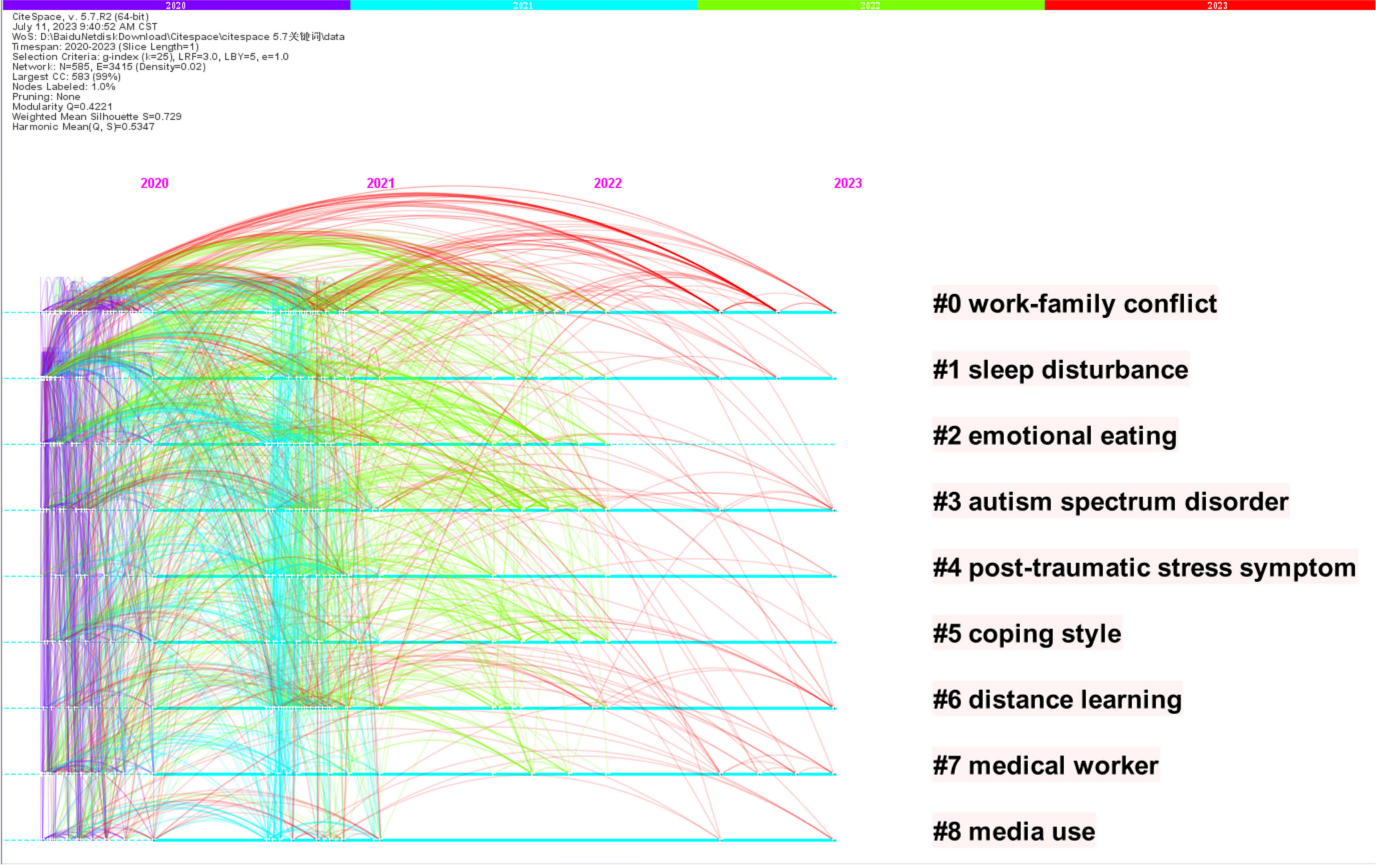
Cluster labels and terms produced between 2020 and 2023.

For analyzing collaborative relationships and critical topics, this study relied on the data processed by the COOC software and used the VOS viewer to map the collaborative networks of nations, institutions, and authors, as well as keyword clustering maps. This study equally analyzed the literature descriptively by counting the quantity of publications, authors, nations, institutions, cited literature, as well as authors’ keywords. In particular, the data restriction for the mapping was countries with more than 5 publications, institutions with more than 15 publications, authors with more than 5 publications, and author keywords with more than 10 occurrences. Furthermore, the pre-processed WOS data was imported into Cite Space and analyzed in order to report clustering labels. In **Figure 2**, 9 clustering labels were generated using Cite Space, with the largest cluster being work-family conflict (#0), active from 2020 to 2023. The second largest cluster was sleep disorders (#1), active from 2020 to 2023. The third to tenth largest clusters were emotional eating (#2), autism spectrum disorder (#3), post-traumatic stress disorder (#4), coping styles (#5), distance learning (#6), healthcare workers (#7), and media use (#8).

## 3 Results

### 3.1 Documentation developments

**Figure 3:**
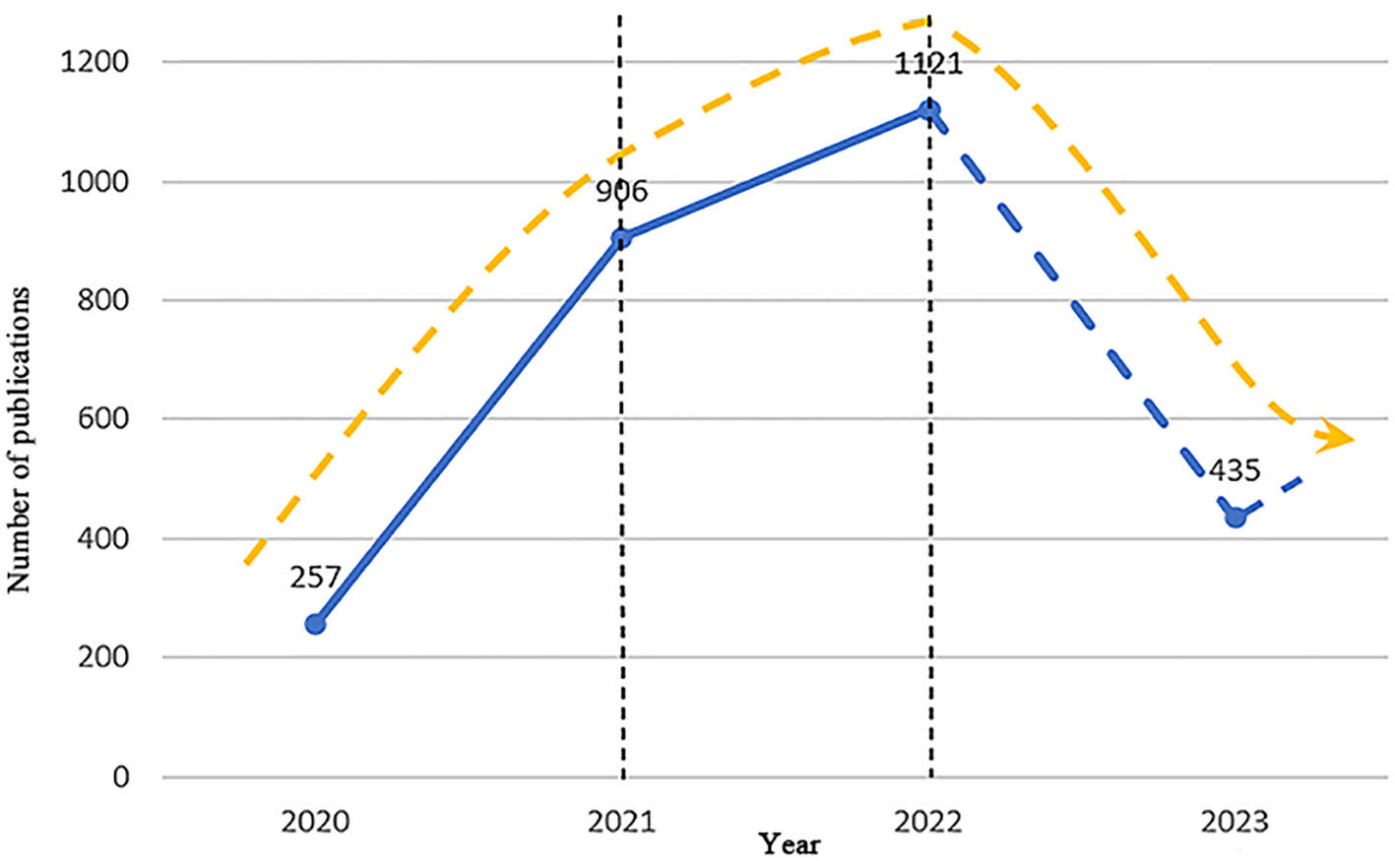
Number of publications.

The valid sample selected for this study consisted of 2,719 articles with 13,502 authors affiliated with 4,028 institutions in 115 countries, citing 92,558 references 5,256 author keywords. From **Figure 3**, the growth in the quantity of papers related to stress during the COVID-19 period can be divided into three phases: the phase of rapid growth, the phase of stable growth, and the phase of quality enhancement. A phase of rapid growth occurred between 2020 and 2021, with a significant increase in the quantity of articles published, by a rate of 649 articles in one year. The stable growth phase occurred from 2021 to 2022, with slower growth within the year compared with the previous phase, with an increase rate of 215 articles in one year. The quality improvement phase occurred from 2022 to 2023, with a substantial decrease in the number of articles published in this phase compared to the previous phase, and as of June 15, 2023, 435 articles have been published in this phase.

### 3.2 Analysis of countries, institutions, authors and journals

1. Highly productive countries and partnerships

**Figure 4:**
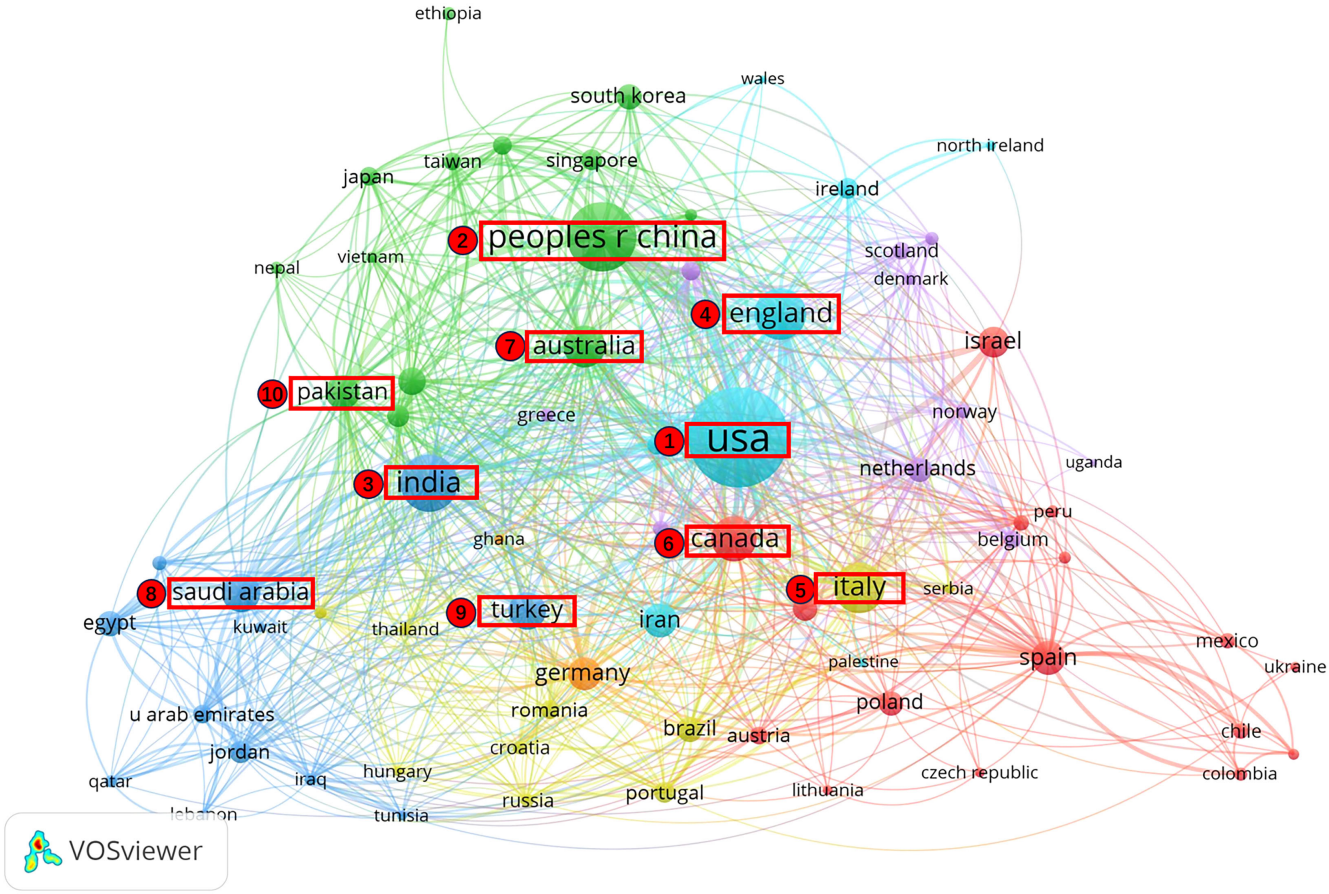
Map of co-countries network.

The study compiled the top ten nations that published the greatest quantity of articles (**Table 1**). Among 115 countries and regions, the USA is the country with the largest number of articles, and it has far more articles than other countries and regions, surpassing China by 374 articles. This indicates that the USA leads in the area of stress during COVID-19. Among the ten high-producing countries and regions, two are located in North America, two in Europe, five in Asia, one in Oceania, with South America and Africa missing on the list. This reveals the low research power in South America and Africa. To further understand the collaborative relationships between countries, this study mapped collaborative relationships (Publication ≥ 5) for 73 countries, as illustrated in **Figure 4**. In the figure, the size of the nodes signifies the quantity of publications, the thickness of the lines signifies the closeness of the cooperation, and different colors signify different sub-network collaboration. Countries in Asia, North America and Europe collaborate closely, while those in South America and Africa do not collaborate closely.

**Table 1:** Most prolific countries/regions ranked by number of publications.

Rank	Country	Publication	Citation	Average Citation/Publication
1	USA	711	9783	13.76
2	Peoples R China	337	4708	13.97
3	India	230	4245	18.46
4	England	190	2954	15.55
5	Italy	179	4047	22.61
6	Canada	148	3787	25.59
7	Australia	127	2511	19.77
8	Saudi Arabia	107	1263	11.8
9	Turkey	98	1961	20.01
10	Pakistan	85	648	7.62

2. Highly productive institutions and collaborative relationships

**Figure 5:**
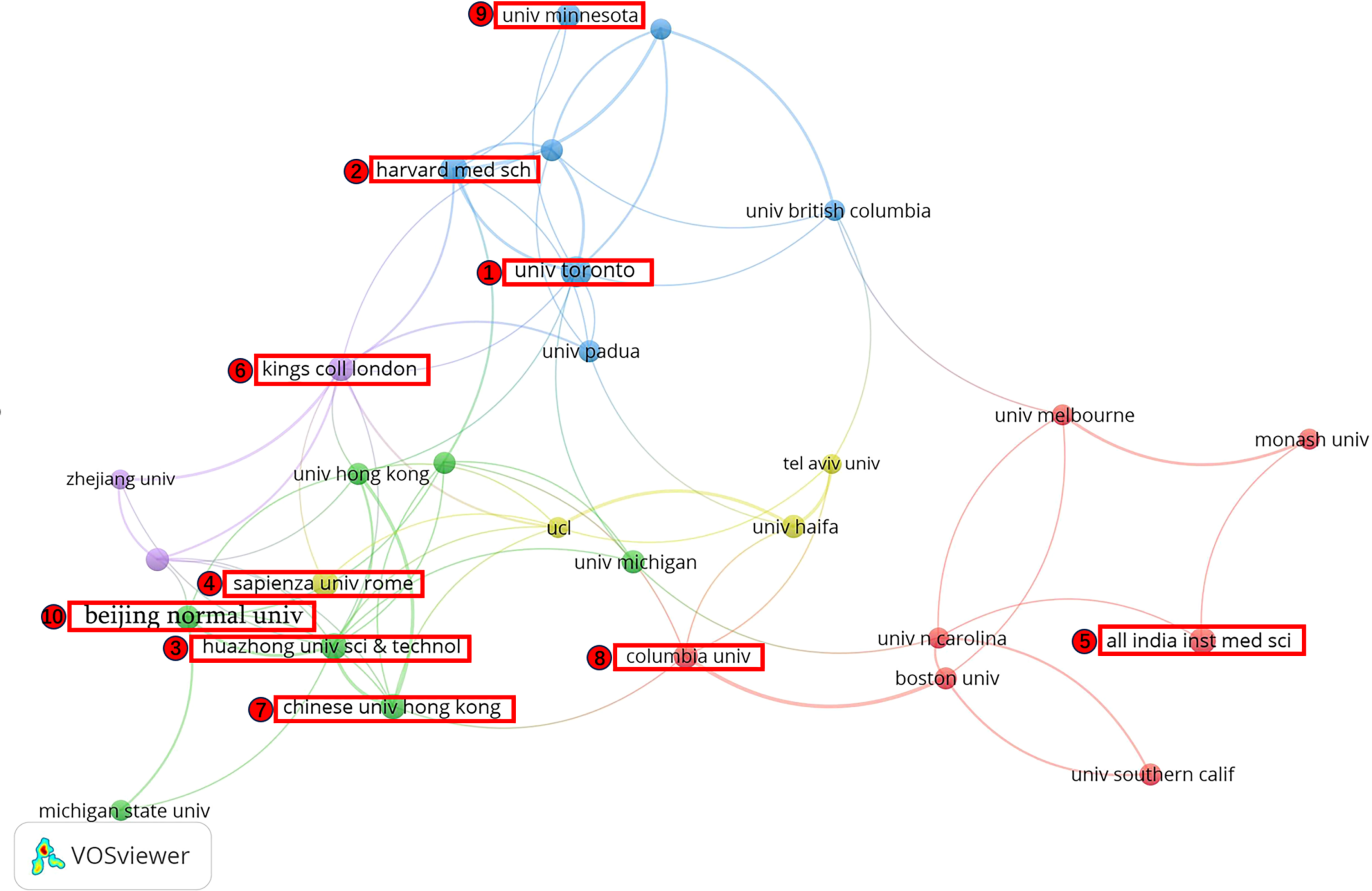
Map of co-institutions network.

In order to analyze the collaborative relationships between institutions in the area of stress during COVID-19, this study mapped institutional collaborations (Publication ≥ 15) and compiled the top ten institutions in terms of publications, as shown in **Table 2** and **Figure 5**. The institutions with publications were the University of Toronto (30 articles) in Canada and Harvard Medical School (25 articles) in the United States. Among them, University of Toronto has shown the highest average citation rate (68.8), which reflects the high quality of its research. Based on the ranking table and the corresponding cooperation chart of institutions, it is obvious that universities were the mainstay of studies on stress during COVID-19. Among which, most are from USA and China. Universities in these countries are more energetic in this field of research, and at the same time there are stronger links between universities in different countries.

**Table 2:** Most prolific institutions ranked by number of publications.

Rank	Institution	Publication	Citation	Average Citation/Publication
1	University of Toronto, Canada	30	2064	68.8
2	Harvard Medical School, USA	25	512	20.48
3	Huazhong University Science & Technology, Peoples R China	23	485	21.09
4	Sapienza University of Rome, Italy	23	155	6.74
5	All India Institute of Medical Sciences, India	23	428	18.61
6	Kings College London, England	22	574	26.09
7	Chinese University Hong Kong, Peoples R China	22	195	8.86
8	Columbia University, USA	20	174	8.7
9	University of Minnesota, USA	20	190	9.5
10	Beijing Normal University, Peoples R China	20	118	5.9

3. Highly productive authors and collaborations

**Figure 6:**
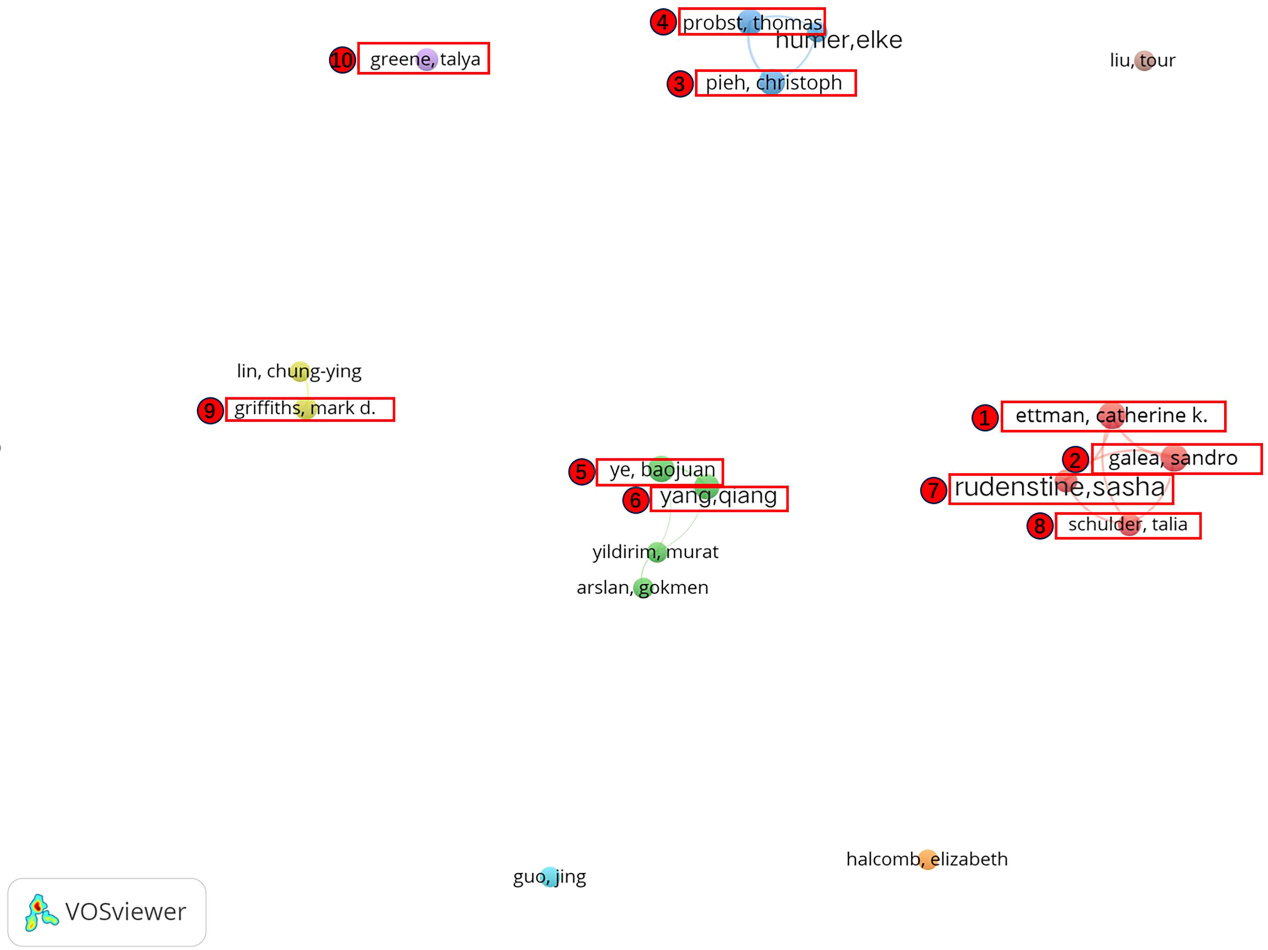
Map of co-authors network.

This study compiled the number of articles, citation frequency, as well as corresponding mean of citations of the top ten high-producing authors in research related to stress during COVID-19 (**Table 3**). Among these, Ettman, Catherine K. and Galea, Sandro published most, both with nine articles. In addition, although Greene, Talya published less, his average citation percentage is higher than that of the other authors, suggesting that his studies are at a high level. Among the top ten highly productive authors, Ettman, Catherine K., Galea, Sandro, Schulder, Talia, and Rudenstine, Sasha collaborated closely and formed a collaborative subnetwork. Moreover, there was a close collaboration between Ye, BaoJuan, and Yang, Qiang; Probst, Thomas, Pieh, Christoph and some other authors formed a collaborative subnetwork. Griffiths, Mark D. and some other authors formed a collaborative subnetwork, while Greene, Talya is relatively independent (**Figure 6**).

**Table 3:** Most prolific authors ranked by number of publications.

Rank	Author	Country	Publication	Citation	Average Citation/Publication
1	Ettman, Catherine K.	USA	9	191	21.22
2	Galea, Sandro	USA	9	191	21.22
3	Pieh, Christoph	Austria	8	216	27
4	Probst, Thomas	Germany	8	216	27
5	Ye, BaoJuan	Peoples R China	8	53	6.63
6	Yang, Qiang	Peoples R China	7	42	6
7	Rudenstine, Sasha	USA	6	32	5.33
8	Schulder, Talia	USA	6	32	5.33
9	Griffiths, Mark D.	UK	6	83	13.83
10	Greene,Talya	Israel	6	193	32.17

4. Analysis of influential journals and cited literature

**Figure 7:**
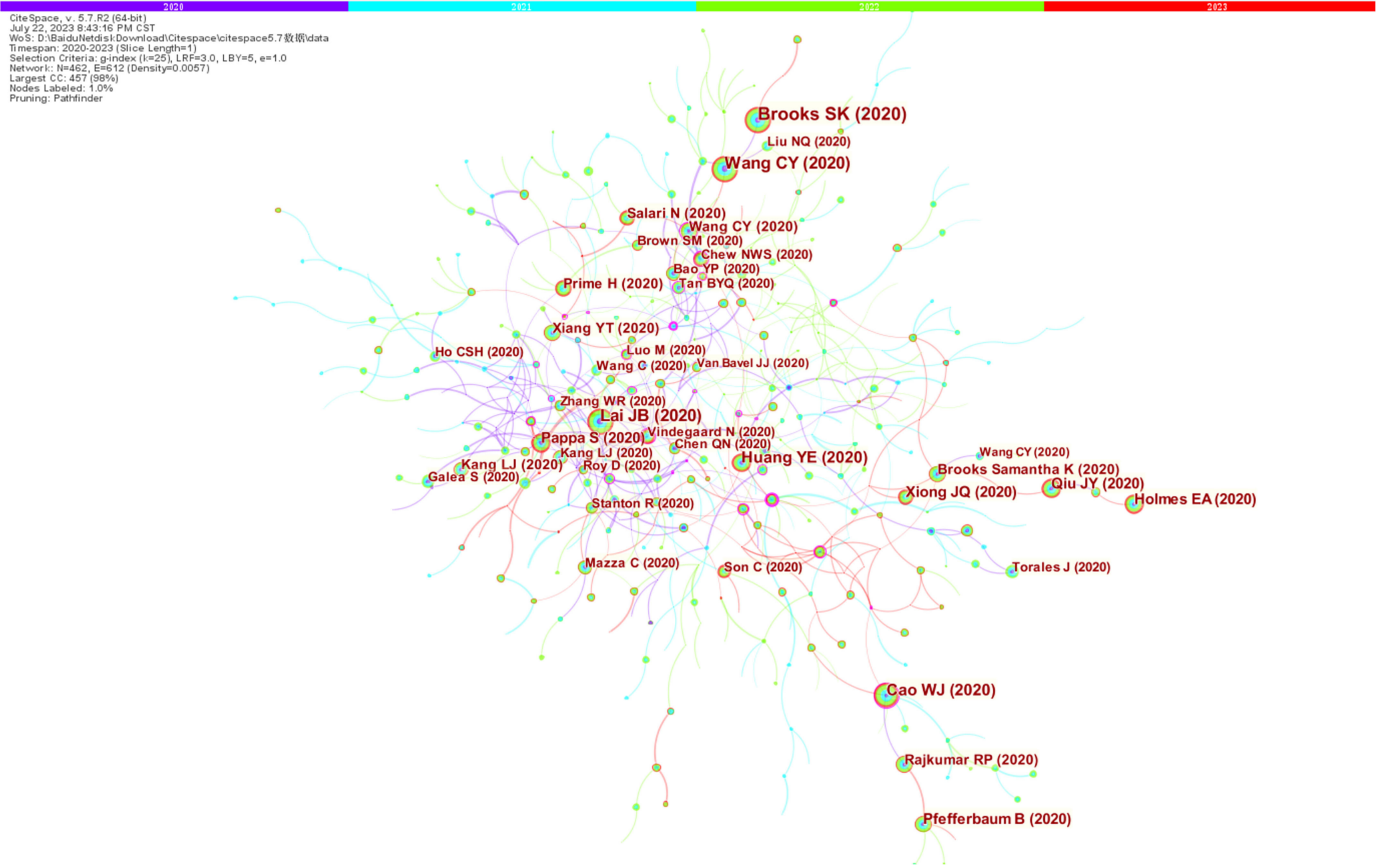
Map of co-cited references.

This study obtained the co-occurrence map of cited literature using Cite Space software (**Figure 7**) and all the records screened were analyzed using VOS viewer software. Four journals are listed in the top ten for both publications and citations, including International Journal of Environmental Research and Public Health, Frontiers in psychology, Frontiers in psychiatry and Journal of affective disorders. It indicates that these journals paid more attention to studies in the area of stress, and they represent the spotlights of research in the field of study (**Table 4**).

**Table 4:** Top 10 influential journals ranked by number of publications.

Rank	Journal	Publication	Citation	Average citation/publication
1	International Journal of Environmental Research and Public Health	165	2895	17.55
2	Frontiers in Psychology	111	1760	15.86
3	Frontiers in Psychiatry	87	959	11.02
4	Frontiers in Public Health	63	353	5.6
5	Healthcare	35	157	4.49
6	Journal of Affective Disorders	32	1447	45.22
7	Current Psychology	30	252	8.4
8	Sustainability	25	404	16.16
9	BMJ Open	23	416	18.09
10	Journal of American College Health	17	159	9.35

From **Table 5**, Wang, cy, Cohen, s, Brooks, sk, Kroenke, k, Lai, jb, Spitzer, rl, and Cao, wj were the most highly cited scholars in the field of stress during the COVID-19 period, and their results were among the top ten most cited literature (Wang cy, 2020; Cohen s, 1983; Brooks sk, 2020; Kroenke k, 2001; Lai jb, 2020; Spitzer rl, 2006; Cao wj, 2020). This reveals that they made a significant contribution to research on stress during COVID-19.

**Table 5:** Top 10 most cited authors and references in “Stress during COVID-19”.

Rank	Cited author	Citation	Rank	Cited reference	Citation
1	World Health, Organization	498	1	Brooks sk (2020)	306
2	Wang, cy	470	2	Lai jb (2020)	252
3	Cohen, s	414	3	Cohen s (1983)	223
4	Brooks, sk	350	4	Wang cy (2020)	217
5	Who	292	5	Spitzer rl (2006)	184
6	Kroenke, k	264	6	Cao wj (2020)	177
7	Lai, jb	252	7	Huang ye (2020)	147
8	Lazarus, rs	228	8	Kroenke k (2001)	134
9	Spitzer, rl	209	9	Qiu jy (2020)	125
10	Cao, wj	177	10	Xiong jq (2020)	125

### 3.3 Thematic analysis

This study used the COOC software to analyze the keywords in the area of stress during COVID-19, and finally gained the top twenty high-frequency author keywords, as listed in **Table 6**. These include COVID-19 (2089 times), stress (509 times), mental health (451 times), anxiety (428 times), depression (397 times), pandemic (264 times), resilience (142 times), healthcare workers (136 times), well-being (106 times), lockdown (99 times), burnout (99 times), and coronavirus (98 times) are more popular in the stress studies during COVID-19. In addition, psychological distress (76 times), social support (71 times), coping (64 times), physical activity (58 times), nurses (57 times), coping strategy (56 times), qualitative research (55 times), and quarantine (54 times) have also gained significant attention.

**Table 6:** High-frequency keywords.

Rank	Keyword	Frequency	Rank	Keyword	Frequency
1	COVID-19	2089	11	BURNOUT	99
2	STRESS	509	12	CORONAVIRUS	98
3	MENTAL HEALTH	451	13	PSYCHOLOGICAL DISTRESS	76
4	ANXIETY	428	14	SOCIAL SUPPORT	71
5	DEPRESSION	397	15	COPING	64
6	PANDEMIC	264	16	PHYSICAL ACTIVITY	58
7	RESILIENCE	142	17	NURSES	57
8	HEALTHCARE WORKERS	136	18	COPING STRATEGY	56
9	WELL-BEING	106	19	QUALITATIVE RESEARCH	55
10	LOCKDOWN	99	20	QUARANTINE	54

**Figure 8:**
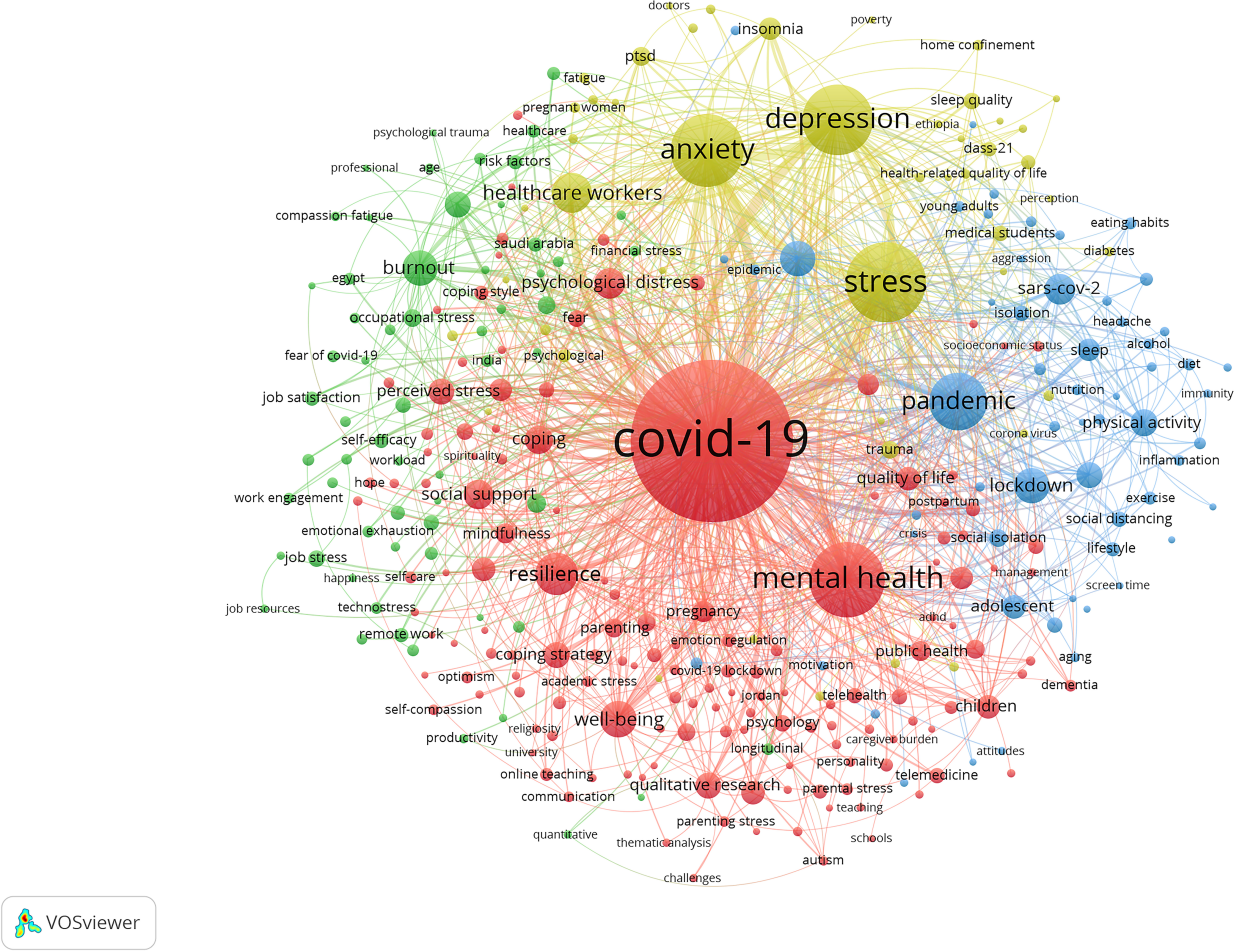
Map of keywords clustering.

In addition, in order to explore the research theme, this study obtained the keywords using the VOS viewer after constructing the keyword co-occurrence matrix using the COOC software and excluding some meaningless keywords, as shown in **Figure 8**. The graph contained 128 items (Frequencies ≥ 10), 1903 links and 4 categories; the degree of the nodes, the strength of the connecting lines, and the number of citations influenced the size of the elements; the colors represented the clusters to which they belong. The topics of greatest interest in the research field were divided into 4 clusters: the influence of stress on the psychological level during COVID-19, the influence of stress on the life level during COVID-19, the influence of stress in the work level during COVID-19, and the influence of stress on the spiritual status during COVID-19.

1. Impact of stress on psychological dimensions during COVID-19 (red)

The major keywords included: COVID-19 (2089 times), mental health (451 times), well-being (106 times), coping strategy (56 times), perceived stress (53 times), children (46 times), students (40 times), loneliness(36 times), psychological stress (26 times), fear (24 times), depressive symptoms (20 times), psychological resilience (15 times), emotional regulation (15 times), emotional distress (12 times), parenting stress (10 times) and so on. This cluster focused on the psychological responses of different population groups, concerned strategies for countering stress during COVID-19, and had an emphasis on people’s mental health.

2. Impact of stress on life dimensions during COVID-19 (green)

The major keywords included: lockdown (99 times), physical activity (58 times), quarantine (54 times), sleep (43 times), social distancing (19 times), lifestyle (18 times), eating habits (11 times), diet (11 times), domestic violence (11 times), and epidemics (10 times). This cluster focuses on the influence of stress generated during COVID-19 on the lifestyle of individuals, as well as the changes during COVID-19 in people’s lifestyles, social interactions and so on.

3. Impact of stress on job level during COVID-19 (blue)

The main keywords included: burnout (99 times), occupational stress (25 times), job stress (23 times), job satisfaction (18 times), self-efficacy (18 times), emotional exhaustion (12 times), turnover intention (11 times), etc. This cluster focuses on the effects of stress during COVID-19 on the working level, and explored ways to alleviate this stress among staff.

4. Impact of stress on mental dimensions during COVID-19 (yellow)

The main keywords included: stress (509 times), anxiety (428 times), depression (397 times), insomnia (41 times), ptsd (31 times), mental well-being (21 times), psychological impact (17 times), post-traumatic stress disorder (14 times). Most words in this cluster are related to the spiritual status of individuals, focusing on the effect of stress during COVID-19, such as anxiety and depression, and exploring measures to mitigate the effects.

### 3.4 Evolutionary trends

**Figure 9:**
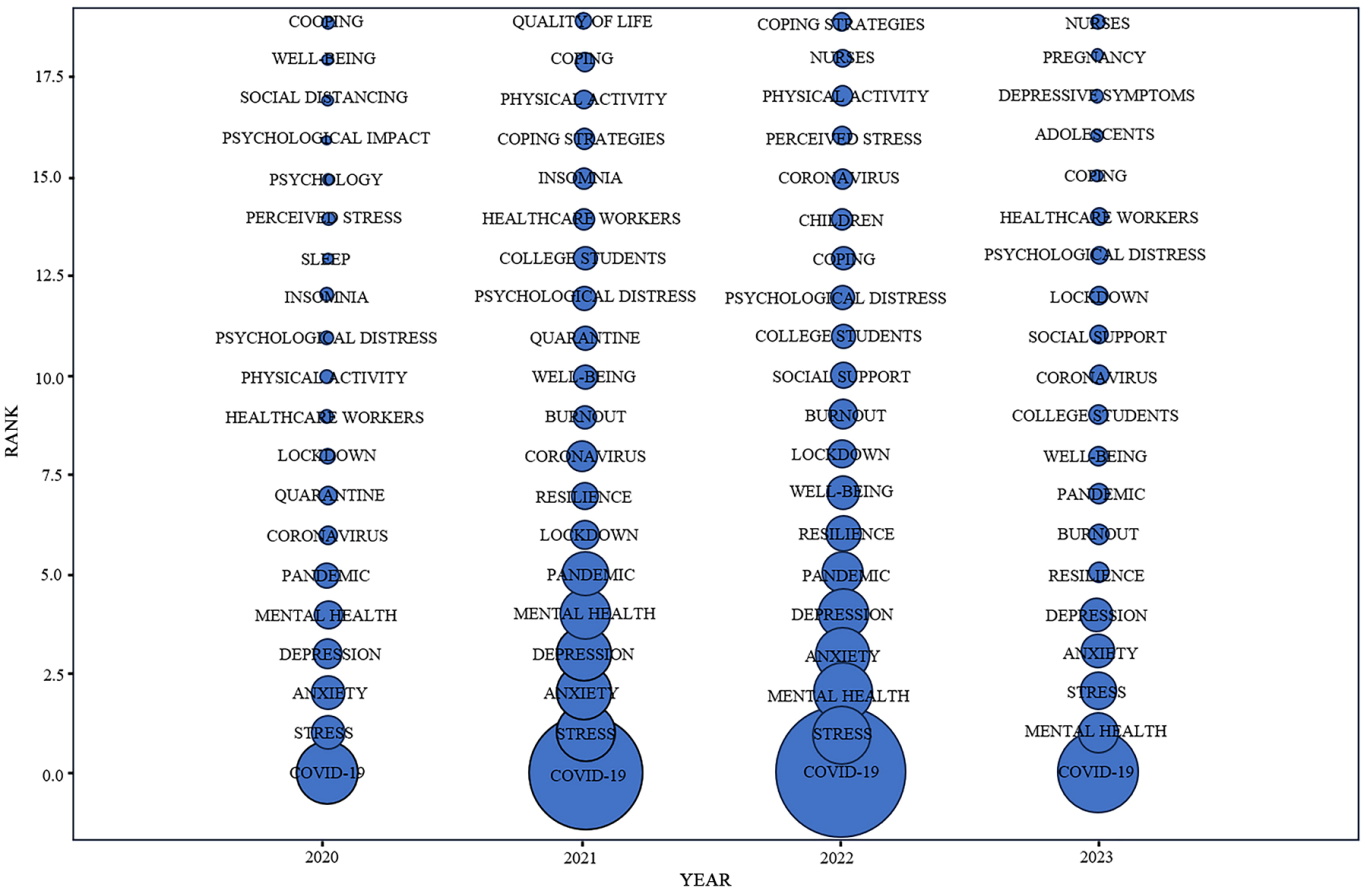
Time zone view of keywords.

In this study, a time zone map of the authors’ keywords was plotted using COOC software (**Figure 9**) to explore the trend of stress studies over time during COVID-19. **Figure 9** illustrates the top 20 author keywords with the highest frequency of occurrence in stress studies during the COVID-19 period, with the circle size representing the frequency of occurrence of the keyword in the corresponding year. The results showed that the evolutionary trend of stress studies during COVID-19 can be categorized into three phases: developmental, exploratory, and evolutionary.

In 2020, studies of stress during COVID-19 concentrated on the types of stress (“Anxiety” 2020; “Depression” 2020 and “Perceived stress” 2020), psychological impact (“Psychological impact” 2020; “Psychological distress”2020 and “Mental health” 2020), and reactions to stress (“Sleep” 2020; “Insomnia” 2020 and “Physical activity” 2020).

From 2021 to 2022, the frequency of each keyword in studies on stress during COVID-19 increased, and the researchers expanded the population and scope. The range of subjects included children, nurses, and college students (“College students” 2021,2022; “Nurses” 2022; “Children” 2022), as well as studies focusing on different types of stress (“Stress” 2021,2022; “Anxiety” 2021,2022; “Mental health” 2021,2022; “Depression” 2021,2022; “Perceived stress” 2022), and emphasizing the handling of stress (“Coping” 2021,2022; “Coping strategies” 2022; “Resilience” 2021,2022).

In 2023, COVID-19, Mental health, and Stress were the top keywords on the list used, suggesting a steady trend of studies on stress during COVID-19. However, since COVID-19 is essentially over after 2022, studies in this phase are no longer limited to discovering the types of stresses during COVID-19, but focus more on the treatment of stresses after COVID-19 (“Coping” 2023; “Resilience “ 2023; and “Social support” 2023).

In summary, studies on stress during COVID-19 continue to evolve and focus more on dealing with stress during COVID-19.

## 4 Discussion

### 4.1 Discussion of results

This paper presents a comprehensive review of stress research during COVID-19 using visualization and scientometric methods. After analyzing 2719 articles on WOS by using COOC, VOS viewer and Cite Space, we ascertained (1): trends in publications (2); high-producing countries, institutions, and authors, as well as collaborations between institutions and authors (3); important journals and cited literature (4); topics of greatest interest; and (5) trends in research area. All discoveries in this study were rooted in the analysis results described above.

First, the year 2022 can be considered as a turning point in the published literature in the area of stress during COVID-19, as measured by the publication volume. Before 2022, the number of publications in this research area grew rapidly. After 2022, the growth in the published literature slows down. This could be due to the fact that the COVID-19 period rendered people susceptible to stress in the face of a pandemic, thereby justifying the increase in the number of studies on stress during this period (56–58). However, the COVID-19 pandemic essentially ended after 2022, and the number of studies on stress during COVID-19 have declined. Despite this decline, research findings in this area will serve as a reference for countries and regions facing similar major public health events.

Second, by counting the most prolific countries, institutions and authors, and analyzing collaborative relationships, the study discovered that the U.S. is the major research power in the field of stress during COVID-19. First, regarding the most fruitful countries, the U.S. contributes the most to research in this area, with 26% of the all publications, exceeding China’s 12%, and has close collaborations with the next nine countries in the list in a decreasing order. Second, regarding the most prolific institutions, 3 of the top 10 organizations belong to the U.S., but they are not the most productive institutions. In contrast, University of Toronto is the most prolific institution, though Canada ranks sixth in the top ten most prolific countries in terms of publishing volumes. This suggests that the U.S. has many organizations involved in research related to this field, whereas Canada’s research efforts are more concentrated in a single institution. In addition, all ten institutions have close collaborative relationships. Finally, regarding the most prolific authors, there are four of the top ten most productive authors from the United States. Ettman, Catherine K. and Galea, Sandro are those with the most publications. In addition, all ten prolific authors have formed their own stable research teams and research directions. It is possible that authors communicated less with other author groups outside of their own collaborative subnetwork due to outbreak closure and geographic location constraints during COVID-19.

Third, this study identified important journals and cited literature in this research area by counting highly productive journals, highly cited authors and literature. First, in terms of high-yield journals, International Journal of Environmental Research and Public Health, Frontiers in Psychology, Frontiers in Psychiatry and Journal of affective disorders—four journals listed in the top ten in terms of publications and citations—are the leading journals in this area of stress during COVID-19. Second, for highly cited authors and cited literature, Wang, cy, Cohen, s, Brooks, sk, Kroenke, k, Lai, jb, Spitzer, rl, and Cao, wj were among the top ten in the number of citations and outcomes cited. Among the top ten cited frequency literature, two were studies on stress, including psychological stress and perceived stress (cao wj, 2020, cohen s, 1983); two were studies on psychological effects and psychological distress during COVID-19 (brooks sk, 2020, qiu jy, 2020); four were studies on psychological health and mental resilience during COVID-19 (huang ye, 2020, lai jb, 2020, wang cy, 2020, xiong jq, 2020); two were studies on psychiatric disorders, including anxiety disorders, depression (kroenke k, 2001, spitzer rl, 2006).

Fourth, COVID-19, Stress, Mental health, Anxiety, and Depression featured most as keywords. This indicates that studies on stress during COVID-19 focused more on stress and related conditions, such as anxiety, and depression, while emphasizing on optimizing mental health (9, 59–61). Through cluster analysis, this study summarized four themes of focus for studies on stress during COVID-19. First, the influence of stress on the psychological dimension during COVID-19. Here, the pandemic forced changes in daily life and shrunk social space leading to different forms of stress (psychological, perceived and parenting stresses). This gives the possibility for interventions to alleviate stress, thereby optimizing mental health for different population groups (healthcare workers, manufacturing workers and pregnant women) during the pandemic (62–67). Second, the influence of stress on the life dimension during COVID-19. COVID-19 quarantine imposed restrictions on activities, created a social distance, and incited stress affecting daily habits and lifestyles (68, 69). In addition, these lifestyle changes differed with genders (70–72) and affected the treatment of other diseases (41, 73–76). Third, the influence of stress on the work dimension during COVID-19. The COVID-19 outbreak changed work patterns of all working groups (teachers, healthcare workers and hotel staff) to varying degrees (77, 78), forcing people to shift their workplaces to their homes. This affected job satisfaction negatively and added work stress (79–81). This could be mitigated by improving the working environment and engaging in fitness exercise (82–85). Moreover, COVID-19 impacted the work of healthcare workers, leading to increased work pressure, and burnout. This could be mitigated by developing stress resilience to ensure optimal physical and mental health (25, 86–88). Fourth, the influence of stress on the spiritual dimension during COVID-19. In the face of stress since from the COVID-19 outbreak, people were highly susceptible to anxiety and frustration (89–92). In addition, the higher the perceived threat from stress or from news in the number of people around them infected with COVID-19, the higher the chance of becoming anxious and depressed, with healthcare workers being the most affected (93–96).

Fifth, the evolution of the area of stress research during COVID-19 can be divided into three phases: developmental, exploratory, and evolving. At the developmental stage, scholars focus on the study and concentrate more on the types of stress and responses from those stressed. This is because of the forced change in daily life (97–99). The exploratory phase involves increasing the quantity of published studies and broadening the scope and target audience while focusing on the types of stress in the pandemic (100). Finally, in the evolving phase, there is a decreasing trend in the growth of the volume of publications, an increase in quality, a greater focus on the management of stress and the maintenance of optimal mental health. This might be due to the fact that the COVID-19 epidemic is essentially over after 2022, and more attention is given to methods of stress relief and mental health maintenance.

### 4.2 Implications

The importance of this research is expressed in three areas. First, in this study, scientific bibliometric tools (COOC, VOS viewer and Cite Space) were utilized to reveal the research structure, hot spots and evolving trends in the research area of stress during COVID-19, in order to deliver accurate and comprehensive information about this field. Second, by analyzing journals, countries, institutions, and authors, this study revealed the current status of research in this field of stress during COVID-19, which can help scholars to rapidly understand the basics of this field and locate target journals or publications. Third, using bibliometric methods, clustering and predicting stress during COVID-19 helps researchers accurately capture the research hot spots in the field and offers ideas for future research.

### 4.3 Limitations and directions for future research

This study deduced a comprehensive framework of studies on stress during the pandemic, despite some limitations we encountered in the process. First, the study sample was drawn from the WOS core collection, which is highly representative, but it does not cover all papers in this research field of stress during the COVID-19 period. We recommend that future endeavors should track literature in 2023 and broaden data sources, such as EBSCO and PubMed. In addition, while bibliometric analyses using specialized software are objective, interpretation of results are subjective. However, we overcame this through several conversations among multiple authors. We will continue to track, create and adopt more interpretive and stipulative models in the future.

## 5 Conclusion

First, this research found that the heat of stress research during COVID-19 has declined, and the main research forces come from the United States and China. In addition, Countries in Asia, North America and Europe collaborate closely. Institutions have close cooperation with each other and authors have formed their own collaborative networks. Second, in terms of research hot spots, the research area of stress during the COVID-19 period has paid particular attention to the influence of stress on the psychological, spiritual, job and life levels, and gradually focuses on stress management strategies. In total, this study of stress during COVID-19 informs future exploration of stress in the setting of similar public health events and provides insight into the direction of subsequent research.

## Data availability statement

The raw data supporting the conclusions of this article will be made available by the authors, without undue reservation.

## Author contributions

LL: Conceptualization, Validation, Visualization, Writing – original draft. GL: Conceptualization, Validation, Visualization, Writing – original draft. YX: Methodology, Writing – original draft, Writing – review & editing. JJ: Formal Analysis, Visualization, Writing – original draft. ZW: Formal Analysis, Visualization, Writing – original draft.
